# Witch Hazel Significantly Improves the Efficacy of Commercially Available Teat Dips

**DOI:** 10.3390/pathogens9020092

**Published:** 2020-02-01

**Authors:** Reuven Rasooly, Adel Molnar, Paula Do, Gianluca Morroni, Lucia Brescini, Oscar Cirioni, Andrea Giacometti, Emmanouil Apostolidis

**Affiliations:** 1 U.S. Department of Agriculture, Agricultural Research Service, Albany, CA 94710, USA; paula.do@usda.gov; 2 Department of Chemistry and Food Science, Framingham State University, Framingham, MA 002185, USA; adelmmolnar@gmail.com; 3 Department of Biomedical Sciences and Public Health, Marche Polytechnic University, 60121 Ancona, Italy; g.morroni@pm.univpm.it (G.M.); lucia.brescini@ospedaliriuniti.marche.it (L.B.); oscar.cirioni@ospedaliriuniti.marche.it (O.C.); a.giacometti@staff.univpm.it (A.G.)

**Keywords:** mastitis, witch hazel extract, biofilm, staphylococcus, *Escherichia coli*, *Pseudomonas aeruginosa*

## Abstract

Bovine intramammary infections (IMIs) are the main cause of economic loss in milk production. Antibiotics are often ineffective in treating infections due to antimicrobial resistance and the formation of bacterial biofilms that enhance bacterial survival and persistence. Teat dips containing germicides are recommended to prevent new IMIs and improve udder health and milk quality. IMIs are often caused by staphylococci, which are Gram-positive bacteria that become pathogenic by forming biofilms and producing toxins. As a model for a teat dip (DIP), the BacStop iodine-based teat dip (DIP) was used. Witch hazel extract (whISOBAX (WH)) was tested because it contains a high concentration of the anti-biofilm/anti-toxin phenolic compound hamamelitannin. We found that the minimal inhibitory or bactericidal concentrations of DIP against planktonic *S. epidermidis* cells increased up to 160-fold in the presence of WH, and that DIP was 10-fold less effective against biofilm cells. While both DIP and WH are effective in inhibiting the growth of *S. aureus*, only WH inhibits toxin production (tested for enterotoxin-A). Importantly, WH also significantly enhances the antibacterial effect of DIP against Gram-negative bacteria that can cause IMIs, like *Escherichia coli* and *Pseudomonas aeruginosa*. Put together, these results suggest that the antibacterial activity of DIP combined with WH is significantly higher, and thus have potential in eradicating bacterial infections, both in acute (planktonic-associated) and in chronic (biofilm-associated) conditions.

## 1. Introduction

Mastitis is the most prevalent and expensive disease of dairy cattle worldwide, costing the U.S. dairy industry about USD 1.7–2 billion annually or 11% of total U.S. milk production [1,2]. The fundamental principle of mastitis control is that the disease is prevented by either decreasing the exposure of the teat ends to potential pathogens or by increasing resistance of dairy cows to infection [3]. However, even with the best prevention methods, bacterial colonization and infection are still a problem.

The most common mastitis pathogens are found either in the udder (contagious pathogens like Staphylococci and *Escherichia coli*) or the cow’s surroundings (environmental pathogens like *Pseudomonas aeruginosa*). Pathogens can spread from infected udders to “clean” udders during the milking process [2]. Once established, many of these infections persist for entire lactations or the life of the cow. Detection is best done by the examination of milk for somatic cell counts (SCCs) (predominantly neutrophils). SCCs are positively correlated with the presence of infection. Inflammatory changes and decreases in milk quality may start with SCCs as low as 100,000 cells/mL [4].

*Staphylococcus aureus* is a Gram-positive contagious bacterial pathogen and is one of the most frequent pathogens causing subclinical and clinical bovine mastitis in the US, and its herd prevalence ranges from 5% to 50% [5]. The coagulase-negative Staphylococci (CNS) species, like *S. epidermidis* have more recently emerged as a relevant mastitis-causing pathogen [6,7]. These bacteria are the most common pathogens recovered from heifer mastitis and, in dairy herds, were recovered from >25% of the herds [1,2]. Although the level of the cow’s immunological response to CNS infection (as determined by SCC) is moderate in comparison to the response to *S. aureus*, the resultant elevated SCC contributes to reduced milk quality and an overall decrease in milk production for the lactation period. Therefore, CNS, not previously considered as significant infectious agents of the mammary gland, are now considered as detrimental to milk quality. CNS are also involved in udder infections, and their persistence is similar to that of *S. aureus* [6,7].

It is not unusual to find dairy herds in which 40%–50% of lactating cows have two or more staph-infected quarters. Cows that have been infected at least once have a greater probability of becoming re-infected. In addition, the probability that a cow may become infected increases with age and with increasing days in milk production [5]. Recurrent infections are often associated with biofilm growth of bacteria, resulting in a loss of sensitivity to antimicrobials and the persistence of infection [8]. 

Staphylococcal pathogenesis is regulated by quorum sensing systems that control bacterial toxin production, stress response, and biofilm formation [9,10,11,12]. The biofilm-forming species, like *S. epidermidis* and the toxin-producing *S. aureus* are notoriously difficult to treat with antibiotics as they facilitate their persistence in the host, evade host defenses, and allow bacterial survival even at high concentrations of antimicrobials [10]. 

Hamamelitannin, a natural active component of witch hazel [13], has been shown to be a quorum sensing inhibitor, interfering with staphylococcal pathogenesis by inhibiting key molecular mechanisms responsible for bacterial stress response, toxin production, and biofilm formation [14]. In the presence of hamamelitannin, staphylococci thus become more vulnerable to host defense mechanisms and antibiotics, making bacterial infections easier to treat with commonly used antibiotics or germicides [14,15,16,17]. Hamamelitannin (2,5-di-O-galloyl-hamamelose) is a phenolic compound that has also been associated with various other health benefits, such as protection from colon cancer [18]. 

Witch hazel (*Hamamelis virginiana* L.) is a deciduous shrub or small tree native in the Northeast USA and Canada. Witch hazel bark extract is widely used as an ingredient in products for the treatment of dermatological problems, and to promote wound healing [19,20]. While the major component of witch hazel bark extract is hamamelitannin, other phenolic compounds are also present, such as gallic acid, gallocatechin, and epigallocatechin [13]. Various reports have demonstrated the antimicrobial potential of gallic acid, gallocatechin, and epigallocatechin against bacterial pathogens, including *Staph. Spp.* [21,22,23].

Prevention of colonization and subsequent infection is important as the treatment of biofilm-forming pathogens is quite difficult. Pre- and post-milking germicidal teat dipping is an effective management practice to prevent transmission of new infections. The most common teat dips (65% of the market) contain iodine [24,25], and others contain germicidal like chlorine dioxide, chlorhexidine, hydrogen peroxide, or sodium hypochlorite [26,27]. But even with the use of these products, it is not unusual to find dairy herds in which 40%–50% or more of lactating cows have two or more quarters infected with staphylococci [5], probably because of the presence of bacterial biofilms that can be up to 1000 times more resistant to antimicrobials [28], making some teat dips ineffective once a biofilm is formed.

Our aim is to develop pre-and post-milking teat dips that would be more effective against both planktonic and biofilm bacteria. Commercially available teat dips were tested together with witch hazel bark extract that contains a high level of hamamelitannin (whISOBAX, StaphOff Biotech Inc). Efficacy studies were carried out on Gram-positive bacteria *S. aureus* and *S. epidermidis* as well as on the gram-negative bacteria *E. coli* and *P. aeruginosa*. 

The development of effective methods of preventing bacterial colonization and consequent mastitis is extremely desirable, leading to reduced costs, improved animal health and milk quality, increased dairy profitability, and increased food safety. 

## 2. Results

*S. epidermidis* is a common producer of biofilms and is a common cause of subclinical cow mastitis [6,7]. *S. aureus* is a toxin producer and is a common cause of clinical mastitis [5,29,30]. Teat dips containing iodine are commonly used before and after milking to prevent such infections [25,27], but the problem of subclinical and clinical mastitis is still prevalent [1,2,3,5]. Therefore, to enhance the antibacterial activity of iodine, we added witch hazel extract that contains high levels of hamamelitannin (HAMA), because of its known anti-biofilm properties [14]. The witch hazel extract used (whISOBAX, StaphOff Biotech Inc) (WH) has 50 mg/mL total dry weight (35% of that is due to HAMA), and a total phenolic content of 12.66 mg/mL GAE (76% of that is due to HAMA) [31]. DIP and WH were tested for their antibacterial activity.

### 2.1. Antibacterial Activity against Planktonic S. epidermidis

**Figure 1:**
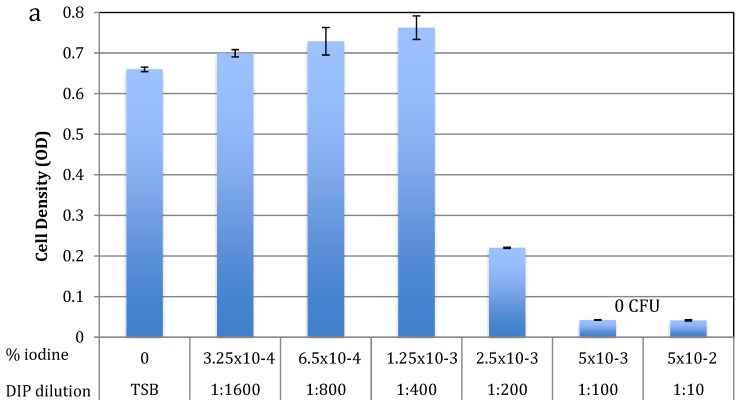

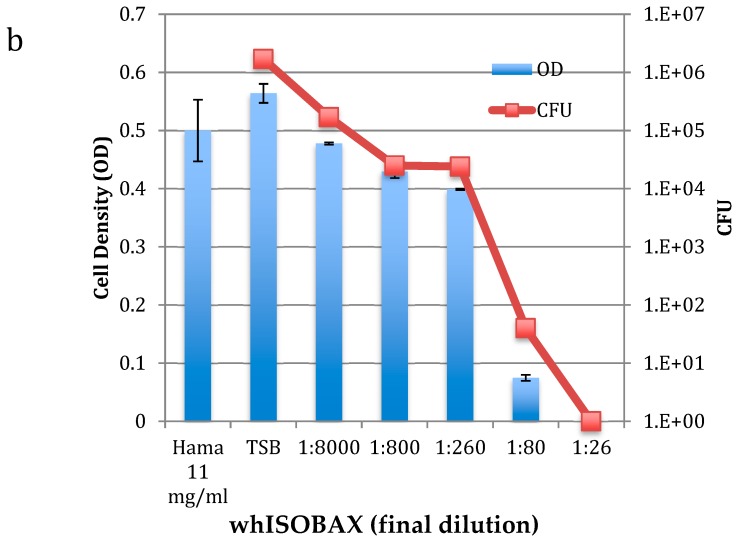
Iodine-based dip BacStop (DIP) (**a**), and witch hazel extract whISOBAX (WH) (**b**) inhibits the growth of planktonic *S. epidermidis* cells. *S. epidermidis* were grown overnight at 37 °C with increasing concentrations of DIP (**a**), WH or HAMA (11 mg/mL) (**b**). Cell density was determined spectrophotometrically at OD 630 nm. Tested dilutions and their respective % iodine are indicated. As a control, cells were grown in TSB culture broth only. Experiments were done in triplicates and standard deviations presented.

To test for antibacterial activity against *S. epidermidis*, early exponential *S. epidermidis* cells were grown overnight with increasing amounts of DIP or WH, and MIC and MBC determined using spectrophotometric and plating methods. As shown in Figure 1a, the MIC of DIP was at 1:200 dilution (>2.5 × 10^−3^ % free iodine) and the MBC was at 1:100 dilution (>5 × 10^−3^ % free iodine). As shown in Figure 1b, the MIC of WH was at 1:80 dilution (containing 0.158 mg/mL GAE and 0.216 mg/mL HAMA) and MBC as 1:26 dilution (containing 0.48 mg/mL GAE and 0.665 mg/mL HAMA). As expected from its known molecular mechanisms [14], HAMA itself does not have bactericidal activities even when tested at high concentrations of 11 mg/mL (Figure 1b). The antibacterial activity observed in WH is thus due to other molecules present, such as the phenolic compounds reported in witch hazel (gallic acid and catechins), which are known to have antibacterial activities [21,22,32]. 

**Figure 2:**
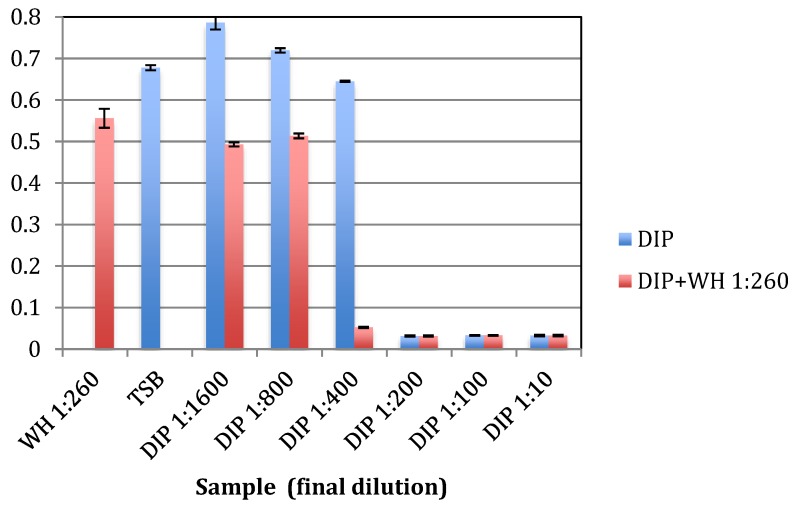
WH + DIP combination has an increased inhibitory effect on planktonic growth of *S. epidermidis*. *S. epidermidis* were grown overnight at 37 °C with increasing amounts of DIP or DIP with WH diluted 1:260, and cell density determined spectrophotometrically at OD 630 nm. As a control, cells were grown in TSB culture broth or with WH diluted 1:260. Experiments were done in triplicates and standard deviations presented.

To test if a combination of DIP and WH has enhanced antibacterial activity against planktonic cells, *S. epidermidis* were grown with increasing concentrations of DIP together with WH 1:260, which is at 10× below its MBC level. As shown in Figure 2, in the presence of WH, the MIC of DIP was 2-fold lower (dilution 1:400 as compared to 1:200) and its MBC also was 2-fold lower (dilution 1:200 as compared to 1:100). These results indicate that WH and DIP act synergistically on planktonic cells and have enhanced antibacterial activity when combined, even when WH was added at 10x below its MBC levels. 

### 2.2. The Effect of DIP and WH on S. epidermidis Biofilm Formation

**Figure 3:**
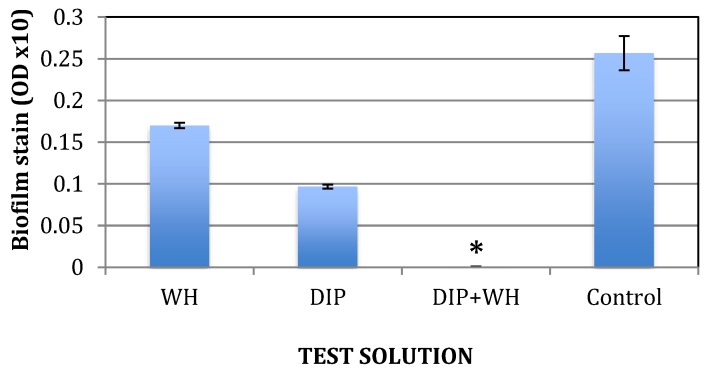
The effect of DIP+/− WH on *S. epidermidis* biofilm formation. *S. epidermidis* were grown for 3 hrs at 37 °C with or without WH (dilution 1:200), DIP (dilution 1:1000), or both. Unbound cells were removed and cell density determined at OD 630 nm. Attached cells were stained by crystal violet, dissolved, diluted 1:10 in water, and OD determined. Zero CFU is indicated by a star. Experiments were done in triplicates and standard deviations presented.

The effect of DIP and WH were tested on the formation of a biofilm by incubating the cells with test solutions for 3 h at 37 °C without shaking and then staining adherent cells. The amount of each test solution was below their respective MBC level. DIP was used at 1:1000 and WH at 1:200 dilutions. The control solution was culture broth (TSB) only. As shown in Figure 3, when DIP and WH were mixed, the formation of a biofilm was abolished (indicated by a star), suggesting an enhanced effect between the two on preventing biofilms from forming. 

### 2.3. The Effect of DIP and WH on Pre-formed Biofilms

**Figure 4:**
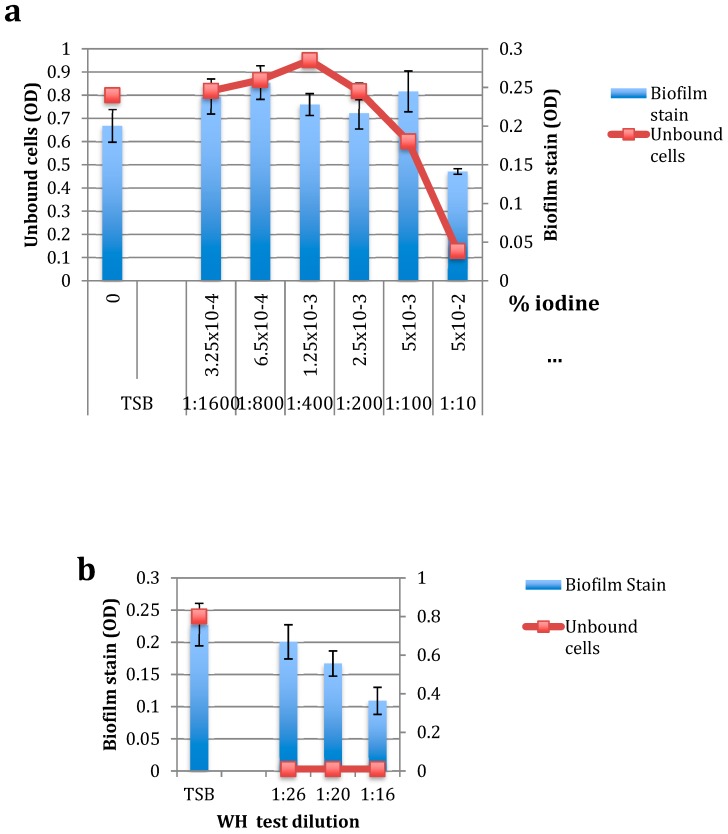
The effect of DIP (**a**) and WH (**b**) on preformed biofilm. Biofilms were formed by growing *S. epidermidis* cells in 96-well plates for several hours to create a detectable biofilm (~5 × 10^6^ CFU). Unbound cells were removed, increasing concentrations of DIP (0–5 × 10^−2^ % iodine) or WH (0–1:26) were added to adherent bacteria, and cells were grown for additional 18 h at 37 °C without shaking. Unbound cells (“UNBOUND”) were removed, and their cell density determined at OD 630 nm. Attached (biofilm) bacteria were stained with crystal violet, solubilized by SDS, and OD determined. Values of initial biofilms were 0.118 OD, 6 × 10^6^ CFU. Experiments were done in triplicates and standard deviations presented.

To test the effect of DIP and WH on pre-formed biofilms, *S. epidermidis* cells in the early log phase of growth were placed in microtiter polystyrene 96-well plates and grown in static conditions for several hours to create a detectable biofilm (containing 5.76 × 10^6^ CFU). Unbound cells were removed, increasing concentrations of DIP (1:10 to 1:1600) or WH (1:16 to 1:26) were added to adherent bacteria, and cells were grown for an additional 18 h. Unbound cells (“UNBOUND”) were removed, and samples plated to determine the MIC and MBC levels. Biofilm bacteria were stained and OD determined. As shown in Figure 4a, at the highest DIP concentration tested (1:10 dilution, or >5 × 10^−2^ % iodine), no unbound cells were found, but the biofilm load was only slightly reduced. At the MBC level of DIP against planktonic cells (1:100 dilution), DIP had no inhibition of biofilm cells and unbound cell load was only slightly (25%) reduced. These results indicate that while DIP is effective in killing planktonic bacteria, it is not as effective in eradicating bacterial biofilms. Biofilms are commonly found on udders of milking cows [8] and can become a source for new infections. 

**Figure 5:**
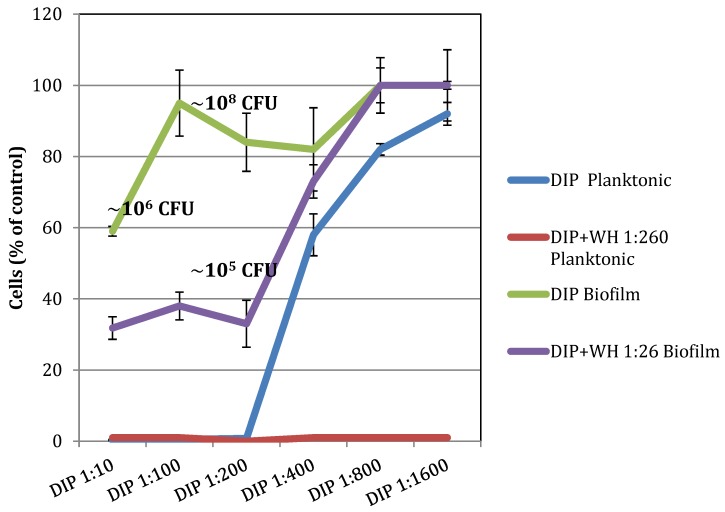
The effect of DIP +/− WH on the growth of planktonic vs. biofilm *S. epidermidis*. *S. epidermidis* (planktonic or biofilm cells) cells were grown with increasing amounts of DIP in the absence or presence of WH 1:260 or WH 1:26. Cell density was determined at OD 630 nm and the amount of biofilm was determined by staining. CFU was determined by plating. Percentages of control values are presented; control (TSB) or WH at the tested dilutions representing 100%. Experiments were done in triplicates and standard deviations presented.

To test for the effect of WH on pre-formed *S. epidermidis* biofilms, WH was added at final dilutions of 1:16–1:26 (at or above MBC levels against planktonic cells). As shown in Figure 4b, WH at 1:16 (containing 1.0 mg/mL HAMA and 0.6 mg/mL GAE) was effective in reducing biofilm load, preventing new biofilm from forming and slightly reducing initial biofilm load. Additionally, no unbound cells were detected, even when WH was diluted to 1:26, which is its MBC level against planktonic cells. These results show that WH is effective against pre-formed biofilms and prevents adherent cells from further growing. The anti-biofilm activity observed in WH is probably due to its high HAMA content, as this tannin is known for its anti-biofilm properties [14,15,16,17]. The effects of DIP and WH on planktonic vs. biofilm *S. epidermidis* are summarized in Figure 5.

### 2.4. The Effect of DIP and WH on S. aureus Growth and Toxin Production 

**Figure 6:**
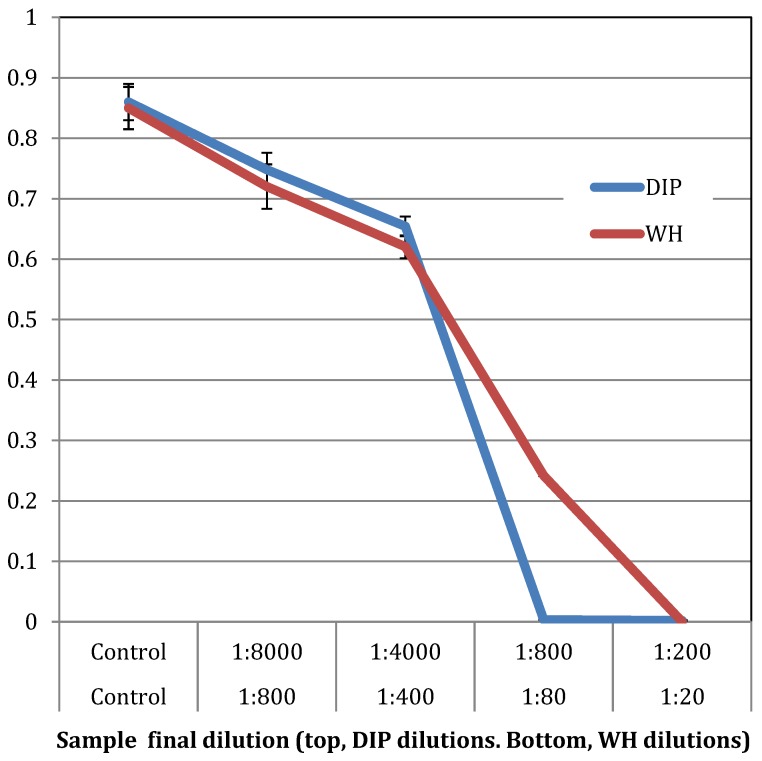
The effect of DIP and WH on the growth of *S. aureus*. *S. aureus cells* (USDA strain) were grown overnight at 37 °C with increasing amounts of DIP or WH, and cell density determined spectrophotometrically at OD 630 nm. Experiments were done in triplicates and standard deviations presented.

The antibacterial effect of DIP and WH were tested on the growth *S. aureus* USDA strain, where early exponential bacteria were grown overnight with increasing concentrations of DIP or WH. As shown in Figure 6, the MBC of DIP is at 1:80 dilution and the MBC of WH is at 1:20 dilution. 

To ensure that DIP and WH also inhibit antibiotic-resistant strains, the MIC tests were carried out on a methicillin-resistant *S. aureus* (MRSA) strain ATCC 43300. The MIC of DIP was shown to be at 1:240, while the MIC of WH was at 1:1920, clearly indicating that the MRSA strains are also sensitive to DIP and WH. 

**Figure 7:**
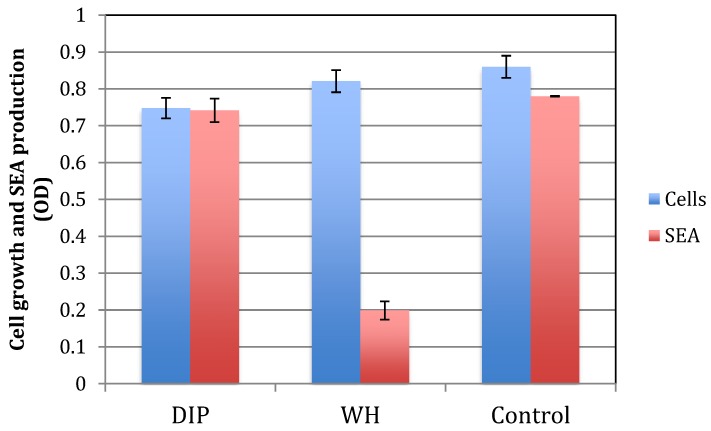
The effect of DIP and WH on *S. aureus* growth and SEA production. *S. aureus* cells were grown overnight with DIP (1:8000), WH (1:800), or TSB as a control. Cell density was determined spectrophotometrically at OD 630 nm (cells). Cells were collected and removed by centrifugation. Supernatants were collected, and the presence of SEA was determined by “sandwich” ELISA (SEA). Experiments were done in triplicates and standard deviations presented.

*S. aureus* produce multiple toxins, which are commonly regulated once their cell number increase and they reach a certain quorum [9,30]. One of these toxins is staphylococcus enterotoxin A (SEA), which is notoriously involved in food poisoning [29]. To test for the effect of DIP or WH on toxin production, we tested for SEA in supernatants of cells grown with DIP or WH. As shown in Figure 7, when DIP or WH was added at dilutions that do not affect cell growth (DIP 1:8000 and WH 1:800), only WH inhibited toxin production. The inhibitory effect of WH is probably due to its high content of HAMA, a known quorum-sensing inhibitor [14]. 

### 2.5. The Effect of DIP and WH on the Growth of Gram-negative Bacteria

To test for the antibacterial activity of DIP and WH on Gram-negative bacteria, DIP and WH were tested on Gram-negative bacteria that can be associated with IMIs, such as *E. coli* and *P. aeruginosa* [33]. *E. coli* are usually associated with transient infections but can cause persistent IMIs through enhanced adherence to host tissue and/or production of shiga-like toxins by certain strains [34,35]. *Pseudomonas* spp., such as *P. aeruginosa*, are environmental mastitis-causing pathogens that spread through the use of water during milking [36]. Checkerboard testing was carried out and fractional inhibitory concentration was calculated to assess the level of synergy between DIP and WH against tested strains. As shown in Table 1, for *E. coli*, the MIC of DIP + WH was two-fold lower than DIP alone (1:160 vs. 1:80 dilution) and 16-fold lower than WH alone (1:160 vs. 1:10 dilution), resulting in an FIC index that is 0.562. Similarly, for *P. aeruginosa*, the MIC of DIP in combination with WH was two-fold lower than DIP alone (1:320 vs. 1:160 dilution) and 8-fold lower than WH alone (1:160 vs. 1:20), resulting in an FIC index that is 0.625. In both cases, these results suggest that combinations of DIP and WH have a significantly enhanced antibacterial effect as compared to each one alone. Since the FIC reflecting synergism is defined as lower than 0.5 [37], we concluded that the effect of DIP and WH is not synergistic but additive. This is not surprising in view of the known molecular mechanisms involved, which differ for WH and DIP [14,32,38,39]. Iodine-based teat dips are bactericidal due to an oxidation-reduction process and by halogenation [38]. WH contains phenolic compounds like gallic acid, gallocatechin, and epigallocatechin that cause bacterial cell disruption by binding to bacterial cell membranes [32,39,40]. A combination of the two can thus have a significantly enhanced antibacterial activity against both Gram-positive and Gram-negative mastitis pathogens. Of note is that the additive effect of the two compounds was confirmed on other bacteria, including various strains of *S. aureus*, where the FIC index is 1 (unpublished).

## 3. Discussion

We show that when tested against planktonic cells, the antibacterial effect of combined DIP and WH is significantly (*p* < 0.05) higher than either one alone (tested on *S. epidermidis*, *S. aureus*, *P. aeruginosa,* and *E. coli*). We also show that DIP is 20-fold less effective against *S. epidermidis* biofilm cells, but the antibiofilm effect is significantly enhanced when mixed with WH. Furthermore, both DIP and WH inhibit the growth of *S. aureus*, but only WH inhibits *S. aureus* toxin production (Table 2 and Table 3). These results indicate the value of using a combination of DIP and WH to eradicate bacterial growth, colonization and pathogenesis, all of which are important factors leading to IMIs. Of note is that similar results were obtained for chlorhexidine-based teat dips and WH, suggesting that the advantage of using a WH combination is not limited to iodine-based teat dips.

Bacteria are increasingly recognized as highly interactive organisms, which is critical to their ability to survive in the host and their capacity to cause disease [11,41]. In particular, many species inhibit biofilms, where they communicate and respond to local cell density through a process known as quorum sensing. Communication occurs through the secretion and detection of autoinducing molecules, which accumulate in a cell density-dependent manner. When the concentrations of the autoinducers reach a threshold level, quorum-sensing cells respond, and genes important for survival are regulated. Quorum sensing and biofilm formation are often closely linked, and it is likely that their interaction is central to the pathogenesis of many bacterial infections [12]. 

Teats are often contaminated by bacteria that adhere to, multiply and within hours, form established biofilms. These biofilms, which are groups of bacteria encased in extracellular matrices, are highly resistant to antimicrobials. Within a biofilm, bacteria can also communicate with one another, activating quorum-sensing systems that lead to the production of numerous toxins, giving an advantage to the bacteria over the host. The toxins produced by *S. aureus* include a family of adhesins that allow bacteria to colonize and form a biofilm [10,30,41]; multiple exotoxins, including toxic-shock syndrome toxin-1, the cause of toxic shock syndrome; enterotoxins that cause food poisoning; proteases that allow the bacteria to spread within the host; and hemolysins, leucocidin, and other virulence factors that affect the outcome of the infective process [9,11,29]. Hamamelitannin has been shown to inhibit staphylococcal pathogenesis (biofilm formation and toxin production) by interfering with stress responses and quorum sensing-based gene regulation, leading to a collapse of the biofilm [14,42]. Bacteria can then be targeted more easily by the host’s immune system and/or by antibiotics, leading to reduced rates on infection, reduced SCC counts, and increased milk quality [43]. 

The antimicrobial effect of DIP and WH is significantly enhanced when the two are combined, reducing bacterial number while preventing bacterial pathogenesis (both biofilm formation and toxin production). Iodine-based teat dips kill bacteria by an oxidation-reduction process and by halogenation [38]. whISOBAX contains high levels of hamamelitannin, that act as a quorum-sensing inhibitor in staphylococci, preventing bacterial toxin production and biofilm formation [14], and was shown both in vitro and in vivo to inhibit bacterial pathogenesis [14,15,16,17]. Hamamelitannin inhibits the phosphorylation of TraP, which is a highly conserved protein among staphylococcal strains and species [43,44,45]. Thus, the effect of hamamelitannin (or WH) is not strain-specific. whISOBAX also contains phenolic compounds such as gallic acid, gallocatechin, and epigallocatechin that cause bacterial cell disruption by binding to bacterial membranes [32,39,40]. While some variation in bacterial sensitivity to these phenolic compounds is found, generally speaking, these compounds are not species- or strain-specific [46]. Indeed, our preliminary studies indicate that WH can inhibit cell growth of various Gram-positive and Gram-negative bacteria, including *Streptococcus agalactiae*, a common mastitis pathogen (unpublished). 

This dual approach reduces bacterial ability to survive in the host and cause disease, eradicating bacterial infections both in acute (planktonic-associated) and in chronic (biofilm-associated) conditions [42], both of which are relevant to udder infections in dairy cows [47,48].

## 4. Materials and Methods

### 4.1. Bacteria

*S. epidermidis* ATCC 35,984 (RP62A), a biofilm-producing strain. *S. aureus* USDA strain, a producer of enterotoxin A (SEA). *S. aureus* ATCC 43300, a methicillin-resistant strain (MRSA). *Escherichia coli* ATCC 25922, *Pseudomonas aeruginosa* ATCC 27853. Staphylococci were grown overnight in Tryptic Soy Broth (TSB) with shaking (220 rpm) at 37 °C, diluted 1:500 in TSB, and grown for about two more hours to the early exponential phase of growth of <0.1 OD_630_ or OD_600_. Gram-negative bacteria were similarly grown in cation-adjusted Mueller–Hinton broth (MH).

### 4.2. Test Formulations

***DIP.*** Iodine-based teat dip (BacStop pre/post Teat Dip, IBA Inc. Millbury, MA) containing 2.5% nonylphenoxypolyethoxyethanol iodine complex, providing 0.5% minimum titratable iodine. 

***WH.*** Witch hazel extract (whISOBAX, 50 mg/mL StaphOff Biotech Inc, MA, USA) containing 50 mg/mL dry weight. Its phenolic content is 12.66 mg/mL gallic-acid equivalent (GAE), where 76% of that is due to hamamelitannin [31]. Dilution factors used in the experiments described are shown in Table 4 and Table 5. Unless noted, chemicals were purchased from Sigma-Aldrich Co. (MO, USA). 

### 4.3. MIC Testing on Planktonic Cells

Minimal inhibitory concentration (MIC) was determined using a microbroth dilution method with an initial inoculum of early exponential bacteria. Cells were grown to the early exponential phase of growth in culture broth and cells (20 µL/well, containing approximately 2 × 10^4^ CFU) were incubated with increasing dilutions of test solutions in a final volume of 200 µL per well (Polystyrene 96-well plates (Falcon, Corning NY)) for about 18 h at 37 °C in air. Cell density was determined using a microtiter plate reader (BioTek Winooski, VT) at optical density of 630 nm (OD630). Cell number was determined by plating samples on Tryptic Soy Agar (TSA) plates, incubating overnight at 37 °C, and colony-forming units (CFU) counted the next day. The MIC was taken as the lowest drug concentration at which observable growth was inhibited. The MBC was taken as the lowest drug concentration that resulted in no bacterial growth. All experiments were performed in triplicates. OD of test solutions in TSB (no cells) were determined and used as background values. Positive controls included growing cells in TBS alone or TSB with relevant solvents (water or ethanol). 

To test for the type of interaction between the compounds, a checkerboard analysis study was used to determine the MIC of a combination of DIP + WH compared to the MIC of each one alone. Checkerboard assays were performed on polypropylene microtiter 96-well plates using cation-adjusted MH broth. These results were used to calculate the fractional inhibitory concentration index (FICI), where the MIC of DIP in combination divided by the MIC of DIP alone + MIC of WH in combination divided by the MIC of WH alone was calculated. FIC <0.5 suggests synergism, while FIC 0.5–4.0 suggests an additive effect [37]. 

### 4.4. MIC Testing on Biofilm Cells 

MIC testing on biofilm cells was carried out essentially as described [49]. *S. epidermidis* were grown in TSB to their early exponential phase of growth (OD 630 nm about 0.045, which is about 1000 CFU/µL). To develop a biofilm, 200 µL were placed in Falcon polystyrene 96-well plates and grown for 4–5 hrs with gentle agitation (50 rpm) at 37 °C. Unbound cells were removed, and bound cells were rinsed 2 times with sterile phosphate buffer saline (PBS) under aseptic conditions. Sample wells were fixed with ethanol to determine the initial biofilm by staining (see below). To adherent cells (about 6 × 10^6^ CFU), 200 µL test solutions (in TSB) were added, and microtiter plates incubated for about 18 h at 37 °C with gentle agitation (50 rpm). Cell density was determined spectrophotometrically at OD 630 nm. Non-adherent cells (“cells”) were removed to another microtiter plate and cell density determined. CFU was determined by plating a sample on TSA plates.

To evaluate the formation of a biofilm, the remaining attached bacteria (“biofilm”) were washed three times with PBS, fixed with ethanol, then the ethanol was removed and cells were air-dried. Biofilm cells were then stained for 5 min with filtered 0.2% crystal violet in 20% ethanol. Unbound stain was rinsed off with water. The plates were air-dried, and the dye bound to adherent cells was solubilized with 200 µL 0.1% SDS. The OD of each well was determined at 630 nm (BioTek Microplate Reader). Tests were performed in triplicates. 

### 4.5. Prevention of Biofilm Formation

Prevention of biofilm formation was carried out essentially as described by [49]. Early exponential cells (150 µL, equivalent to approximately 1.5 × 10^5^ *S. epidermidis*) were placed in polystyrene 96-well plates (Falcon), and test solutions added to a final volume of 200 µL. Cells were grown for 3 hrs without shaking at 37 °C. Unbound cells were then removed, and attached cells (“biofilm”) were gently washed with PBS and stained as described above. 

### 4.6. SEA Production 

To determine the amount of SEA produced by *S. aureus*, ELISA “sandwich” testing was used as described [50]. Sheep anti-SEA IgG (Toxin Technology, Sarasota, FL, USA) was used as the capture antibody, and sheep anti-SEA horse radish peroxidase (HRPO) (Toxin Technology, Sarasota, FL, USA) was used as the detection antibody. The capture antibody was diluted in coating buffer (0.01 M NaHCO_3_, 0.1 M Na_2_CO_3_) at a final concentration of 10 µg/mL, and 100 µL/well was added to microtiter 96-well plates (Greiner, NC, USA) and incubated for 1 hr at 37 °C or overnight at 4 °C. Plates were washed 3 times with PBST (PBS containing 0.05% Tween-20), and the same solution (100 µL/well) was used for blocking unbound sites for 15 min at room temperature (RT). To prepare test samples, treated cells were removed by centrifugation, and supernatants collected. One hundred microliters of each sample were added (in triplicate wells) and plate incubated for 2 h at 37 °C. Plates were washed 3 times with PBST. The detection antibody, diluted 1:300 in PBST, was added (100 µL/well) and incubated for 1 hr at 37 °C. Plates were washed 5 times with PBST. One hundred microliters of 3,3′,5,5-tetramethylbenzidine chromogen solution (Invitrogen, Carlsbad, CA) substrate was added, and 0.3 HCl (50 µL/well) was added to stop the reaction. Absorbance was measured at 450 nm in a microplate reader (BioTek, Winooski, VT) and expressed as 10X OD measured. All tests were performed in triplicate. Increasing amounts of SEA (1 µg/mL to 10 ng/mL) was used as a standard curve. 

### 4.7. Statistical Analysis

All experiments were carried out in triplicates and averages presented. Standard deviation was calculated using the “unbiased“ n-1 method by Microsoft Excel. The significance of differences between treatment groups was calculated using a two-tailed Student’s *t*-test. *p* < 0.05 was considered significant.

## Figures and Tables

**Table 1:** Checkerboard testing of DIP (starting solution of 0.5% iodine) and WH (starting solution of 50 mg/mL). The MIC of each alone and in combination (D–W) is presented and used to calculate FICI.

MIC DIP	MIC WH	MIC Combination (D-W)	FICI	MIC DIP	MIC WH	MIC Combination (D–W)	FICI
*E. coli*				*P. aeruginosa*			
1:80	1:10	1:160/1:160	0.562	1:160	1:20	1:320/1:160	0.625

**Table 2:** Summary of antimicrobial activity of DIP and WH against *S. epidermidis*.

*S. epidermidis*	Planktonic	Planktonic	Biofilm	Biofilm
	MIC Dilution	MBC Dilution	MIC Dilution	MBC Dilution
**whISOBAX**	1:80	1:26	1:26	< 1:16
**DIP**	1:200	1:100	1:10 (up to 40% reduction of biofilm)	
**DIP + WH 1:26**	>1:1600	>1:1600	1:200 (up to 70% reduction of biofilm)	
**DIP + whISOBAX 1:260**	1:400	1:200		
**whISOBAX + DIP 1:100**			1:26	< 1:16

**Table 3:** Summary of antimicrobial activity of DIP and WH against *S. aureus*.

*S. aureus*	MIC	MBC	Inhibition of SEA Production (Max Dilution)
**WH**	1:20	1:40	1:800
**DIP**	1:800	1:1600	No activity

**Table 4:** DIP dilutions and their respective content of active ingredients. The MBCs against planktonic growth of *S. epidermidis* are indicated.

Final Tested Dilution of DIP	% Iodine in Final Dilution
1:10	>5 × 10^−2^
1:100 *	>5 × 10^−3^
1:200 **	>2.5 × 10^−3^
1:400	>1.25 × 10^−3^
1:800	>6.5 × 10^−4^
1:1600	>3.25 × 10^−4^

* MBC of DIP against planktonic cells. ** MBC of DIP against planktonic cells when mixed with WH 1:260.

**Table 5:** WH dilutions and respective content of active ingredients. The MICs against planktonic and biofilm growth of *S. epidermidis* are indicated.

Final Tested Dilution of WH	Final mg/mL GAE (Gallic Acid Equivalent)	Final mg/mL HAMA (Dry Weight Equivalent)
1:16	0.79	1.08
1:20	0.633	0.865
1:26 **	0.48	0.665
1:40	0.31	0.435
1:80 *	0.158	0.216
1:200	0.0633	0.0865
1:400	0.031	0.0435
1:800	0.0158	0.0216

* MIC of WH against planktonic cells; ** MIC of WH against biofilm cells; HAMA, hamamelitannin; GAE, gallic-acid equivalent.
